# Blood pressure measurement using only a smartphone

**DOI:** 10.1038/s41746-022-00629-2

**Published:** 2022-07-06

**Authors:** Lorenz Frey, Carlo Menon, Mohamed Elgendi

**Affiliations:** Biomedical and Mobile Health Technology Lab, ETH Zurich, Zurich, 8008 Switzerland

**Keywords:** Diagnostic markers, Biomedical engineering, Computer science, Hypertension

## Abstract

Hypertension is an immense challenge in public health. As one of the most prevalent medical conditions worldwide, it is a major cause of premature death. At present, the detection, diagnosis and monitoring of hypertension are subject to several limitations. In this review, we conducted a literature search on blood pressure measurement using only a smartphone, which has the potential to overcome current limitations and thus pave the way for long-term ambulatory blood pressure monitoring on a large scale. Among the 333 articles identified, we included 25 relevant articles over the past decade (November 2011–November 2021) and analyzed the described approaches to the types of underlying data recorded with smartphone sensors, the signal processing techniques applied to construct the desired signals, the features extracted from the constructed signals, and the algorithms used to estimate blood pressure. In addition, we analyzed the validation of the proposed methods against reference blood pressure measurements. We further examined and compared the effectiveness of the proposed approaches. Among the 25 articles, 23 propose an approach that requires direct contact between the sensor and the subject and two articles propose a contactless approach based on facial videos. The sample sizes in the identified articles range from three to 3000 subjects, where 8 articles used sample sizes of 85 or more subjects. Furthermore, 10 articles include hypertensive subjects in their participant pools. The methodologies applied for the evaluation of blood pressure measurement accuracy vary considerably among the analyzed articles. There is no consistency regarding the methods for blood pressure data collection and the reference blood pressure measurement and validation. Moreover, no established protocol is currently available for the validation of blood pressure measuring technologies using only a smartphone. We conclude the review with a discussion of the results and with recommendations for future research on the topic.

## Introduction

Hypertension is a serious medical condition and a major cause of premature death. According to the World Health Organization, an estimated 1.28 billion adults aged 30–79 years worldwide have hypertension, with two-thirds of this population living in low- and middle-income countries^1^. The detection, diagnosis and monitoring of elevated blood pressure (BP) remains an immense public health challenge.

Hypertension is typically asymptomatic, which greatly reduces the chances of early detection. BP measurements conducted in a clinical setting over a short time period, are often biased by white coat hypertension, masked hypertension, and similar phenomena, which complicates the detection and diagnosis. Subjects with white coat hypertension exhibit elevated blood pressure values in a clinical setting compared to an ambulatory setting, presumably due to anxiety experienced during a clinical visit^2^. On the other hand, masked hypertension is a phenomenon where BP of patients is normal in the clinic but elevated out of the clinic^3^.

Hypertension can be monitored with various different methods. Invasive (intra-arterial) BP monitoring, commonly used in intensive care units, returns an accurate and continuous BP measurement. However, the need for a trained operator, comparatively high costs, and the time-consuming nature of the procedure render this technique unsuitable for monitoring elevated BP in an ambulatory setting^4^. These difficulties can be overcome with non-invasive, cuff-based BP monitoring devices. A recent study^5^, has clearly shown that many BP monitoring devices (including oscillometric machines and gold standard mercury auscultation) do not accurately represent the BP within the arteries at the upper arm (brachial) or central aorta. Nonetheless, automated oscillometry and mercury auscultation allow for a simpler measurement of systolic and diastolic BP and are therefore predominantly used today. The limiting factors of these devices are the intermittency of measurements, as well as the possibility of erroneous measurements due to movement, inadequate inflation of the cuff, or incorrect cuff size^6^. Furthermore, BP cuffs are obtrusive and uncomfortable to wear during daily activity and are thus not practical for regular measurements throughout the day.

Arterial applanation tonometry and the volume clamp method are other continuous non-invasive BP measurement techniques. However, volume clamping requires a finger cuff, and tonometry is dependent on a cuff-based calibration^7^. Furthermore, both techniques are prone to erroneous measurements due to patient movement^4^. In summary, none of these techniques is well suited for unobtrusive and continuous blood pressure monitoring throughout the day.

Research on BP measurement using only a smartphone has intensified in recent years. Measuring blood pressure with a smartphone would allow for highly cost-effective and unobtrusive monitoring of hypertension. The steadily growing percentage of people owning a smartphone increases the promise of on-the-go monitoring of elevated blood pressure on a large scale. Advances in smartphone technology, especially improved camera and processor performance, are also potential catalysts of progress in this field.

In this review, publications from the last decade dedicated to measuring BP with the use of only a smartphone are identified and examined. In the “Methods” section, we outline the workflow of our literature search. In the results section, we first present an analysis and comparison of the underlying data from all articles in terms of the characteristics of the subjects. Subsequently, we analyze the data types that were recorded and how they were recorded, the signal processing applied to the raw data, the algorithms used to estimate BP from the processed data, and how the proposed methods were validated.

## Methods

**Fig. 1:**
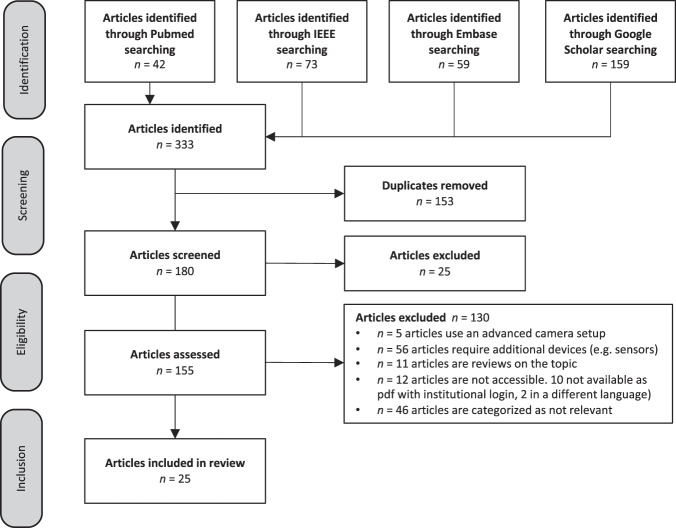
Workflow of the study. Identification, screening, eligibility, and inclusion of articles.

In order to identify as many relevant publications on the topic as possible, we conducted a literature search on four different platforms: IEEE Xplore, PubMed, Embase, and Google Scholar. The search was conducted in accordance with the PRISMA guidelines^8^. Taking advantage of the advanced search filter and with a search base-specific syntax, we were able to define highly specific search terms, thus obtaining a large number of relevant publications and greatly simplifying the subsequent categorization. We restricted our search to the period from November 2011 to November 2021. This allowed us to focus on recent trends and developments in this field. Despite the thorough literature search, we are aware that some relevant publications might have been missed. In the search, we combined the following terms with the logical operators OR and AND: blood pressure, hypertension, image, imaging, face, facial, video, camera, finger, chest, contactless, contact-less, non-contact, noncontact, cuffless, cuff-less, smartphone, and smart-phone. The workflow of the literature search is shown in Fig. 1.

On IEEE Xplore, 73 articles were identified with the following search terms: ("Document Title”: blood pressure OR “Document Title”: hypertension) AND ("Full Text Only”: image OR imaging OR face OR facial OR video OR camera OR finger OR chest OR contactless OR contact-less OR non-contact OR noncontact OR cuffless OR cuff-less) AND ("Full Text Only”: smartphone OR smart-phone). On PubMed, 42 articles were identified with the following search terms: (blood pressure[Title] OR hypertension[Title]) AND (image OR imaging OR face OR facial OR video OR camera OR finger OR chest OR contactless OR contact-less OR non-contact OR noncontact OR cuffless OR cuff-less) AND (smartphone OR smart-phone). On Embase, 59 articles were retrieved using the following search terms: ("hypertension”:ti OR “blood pressure”:ti) AND (image OR imaging OR face OR facial OR video OR camera OR finger OR chest OR contactless OR “contact less” OR “non contact” OR noncontact OR cuffless OR “cuff less”) AND (smartphone OR “smart phone”) AND [2011–2021]/py. We adjusted the search terms for Google Scholar since it offers a very limited advanced search filter and thus search results can reach very large numbers. On Scholar, 159 articles were identified with the following search terms: allintitle: hypertension OR “blood pressure” smartphone OR smart-phone OR “smart phones”. During screening, 153 duplicates and 25 articles that did not constitute a paper in the desired format were removed. From this point, 155 articles were assessed for eligibility. In the next step, we excluded all articles that were not dedicated to measuring BP using only a smartphone. As seen in Fig. 1, five articles that used an advanced camera setup were excluded. Another 56 articles were excluded because additional devices, e.g., sensors or a second smartphone, were used to measure BP. We excluded 11 reviews from our search. We categorized 12 articles as inaccessible, either due to the record being written in another language (*n* = 2) or because the publication is unavailable as a PDF with our institutional login. In addition, 46 articles were categorized as irrelevant, either because measuring BP was not their main focus or they fell under two or more exclusion criteria. After completing all the steps of filtering and excluding articles, 25 articles were included in our detailed analysis.

## Results

This section outlines the findings of our review. First, a brief overview with possible ways of categorization is provided in the subsection on “Publications”. Second, the participant pools for data acquisition are are analyzed. Third, the datatypes measured by a smartphone that are correlated to BP are examined, followed by an analysis of the BP estimation algorithms and the evaluation of the proposed methods.

### Publications

BP measurement conducted with only a smartphone can be categorized under two main groups: contact and contactless, where “contact” indicates that the signal acquisition requires physical contact between the sensor and the subject. In most cases, this means that the subject’s finger is placed on the smartphone camera. Contactless measurement entails a video-based recording of a physiological signal where no direct contact between the camera and measurement site is necessary. In this review, 23 of the articles were on contact-based BP estimation^9–31^, and only two on contactless BP estimation^32,33^. Combining both groups and within our time window of November 2011–November 2021, there was a clear trend toward increased research activity in the field of BP measurement using only a smartphone. Looking at the geographical distribution of publications in this review, Asia led with 14 of 25 articles (seven from India, four from China, two from Japan and one from South Korea), followed by Europe with eight of 25 articles (five from Switzerland, one each from Italy, Germany, and the Czech Republic), and the United States with three articles.

### Number of subjects and gender distribution

The sample size of all articles analyzed in this review ranged between three and 3000 subjects. Six articles used a sample size smaller than 10 subjects^18,24,25,29,31,32^, and 8 articles used sample sizes of 85 or more subjects^9,11,14,16,22,23,28,33^ (Table 1).

**Table 1:** This table summarizes the content of all the publications analyzed in the review.

Publication			Subjects				Data acquisition					Algorithm				Evaluation	
Author (year)	Number of participants (F:M)	BP status	Comorbidities	Age (years) and ethnicity	Sensor (light from anatomical site)	Device (frame rate)	Site	Recording: repetitions × time [s]	Distance [cm]	Environment	Feature types used (number of features)	Demographic information included in model	Calibration	Reference measurement	Bland-Altman, MAE ± SD (mmHg)	Correlation (Pearson *r* or Spearman *ρ*)	Accuracy (%) (MAPD or classification accuracy)
*Contact*																	
Degott et al.^9^ (2021)	91 (49:42)	NTN/HTN/HoTN	Yes	52.9 ± 15.9 N/R	PPG (green) from finger	Samsung Galaxy S7 (N/R)	Finger	N/R	–	N/R	N/R	N/R	Yes	Sphygmomanometer (A) (A&D UM-101)	SBP = 0.5 ± 7.7 DBP = 0.4 ± 4.6 (ME)	N/R	N/R
Yamakoshi et al.^10^ (2021)	13 (7:6)	NTN/HTN	No	19–73 Japanese	PPG (green) from finger	Different models (60 fps)	Finger	20 × 15	–	Indoors (subject’s home)	Normalized pulse volume, pulse rate (2)	No	Yes	Sphygmomanometer (N/R) (DSK-1051, NISSEI)	SBP = 3.50 ± 2.35 DBP = 4.40 ± 3.09 (MAE)	*r*(SBP) = 0.968 *r*(DBP) = 0.844	N/R
Dörr et al.^11^ (2020)	965 (477:488)	NTN/HTN	Yes	51.0 ± 18.9 N/R	PPG (green) from finger	iPhone 4s (30 fps)	Finger	3 × 120	–	Indoors	Time-domain, frequency-domain (N/R)	Yes	No	Sphygmomanometer (O) (Omron-HBP-1300)	SBP = −0.41 ± 16.52 DBP = N/R (ME)	*ρ*(SBP) = 0.426 *ρ*(DBP) = N/R	N/R
Schoettker et al.^12^ (2020)	51 (25:26)	NTN/HTN/HoTN	No	53.9 ± 17.5 N/R	PPG (green) from finger	Samsung Galaxy S7 (30 fps)	Finger	10 × 60 (training) 7 × 120(validation)	–	N/R	Derivative-based (N/R)	Yes	Yes	Sphygmomanometer (A) (A&D UM-101)	SBP = −0.7 ± 7.7 DBP = −0.4 ± 4.5 (ME)	N/R	N/R
Baek et al.^13^ (2020)	26 (N/R)	N/R	N/R	N/R N/R	PPG (green) from finger	Samsung Galaxy Note 8 (100 fps)	Finger	23 × 90	–	N/R	Time-domain, frequency-domain, entropy-based (N/R)	No	No	N/R	SBP = 5.28 ± 1.80 DBP = 4.92 ± 2.42 (MAE)	N/R	N/R
Devaki et al.^14^ (2020)	140 (N/R)	NTN/HTN	N/R	N/R N/R	PPG (red) from finger	Letv le max 2 le ×821 (30 fps)	Finger	1 × 30	–	N/R	Time-domain (15)	No	No	N/R	N/R	N/R	N/R
Nemcova et al.^15^ (2020)	22 (13:9)	N/R	N/R	N/R N/R	PPG (red) from finger and PCG from chest	Samsung Galaxy S7 (30 fps)	Finger and chest	N/R	–	N/R	PTT	Yes	Yes	Sphygmomanometer (A)	SBP = 5.13 ± N/R DBP = 7.53 ± N/R (MAE)	*r*(SBP) = 0.896 *r*(DBP) = N/R	N/R
Dey et al.^16^ (2018)	205 (115:90)	N/R	N/R	39.8 ± 14.8 Diverse	PPG (IR) from finger	Samsung Galaxy S6 (N/R)	Finger	1 × 900	–	N/R	Time-domain, derivative-based, frequency-domain (233)	Yes	No	Sphygmomanometer (A)	SBP = 6.9 ± 9.0 DBP = 5.0 ± 6.1 (MAE)	N/R	N/R
Matsumura et al.^17^ (2018)	13 (6:7)	NTN/HTN	No	20–24 Japanese	PPG (green) from finger	iPhone 6s (60 fps)	Finger	4 × 45	–	Conference room (4 × 5 m)	Heart-rate, normalized pulse volume(2)	No	No	Sphygmomanometer (N/R) (DS-S10, NISSEI)	SBP = 0.67 ± 12.7 DBP = 0.45 ± 8.6 (ME)	*r*(SBP) = 0.685 *r*(DBP) = 0.685	N/R
Wang et al.^18^ (2018)	7 (N/R)	N/R	N/R	44 ± 17 N/R	PPG (N/R) from finger and SCG from chest	Google Pixel phone (30 fps)	Finger and chest	7 × 30	–	Office room	PTT	No	Yes	Sphygmomanometer (O) (Microlife bp3na1−1*x*)	SBP = N/R DBP = 5.2 ± 2.0 (RMSE)	*r*(SBP) = N/R *r*(DBP) = 0.55	N/R
Raichle et al.^19^ (2018)	32 (32:0)	NTN/HTN	Yes	31.6 ± 5.1 N/R	PPG (green) from finger	iPhone 4s (30 fps)	Finger	3 × 120	–	N/R	Time-domain, frequency-domain (N/R)	Yes	No	Sphygmomanometer (O) (OMRON HBP1300)	SBP = 5.0 ± 14.50 DBP = N/R (ME)	*ρ*(SBP) = 0.401 *ρ*(DBP) = N/R	N/R
Datta et al.^20^ (2017)	50 (N/R)	N/R	N/R	45 ± 17 N/R	PPG (red) from finger	Nexus 5 (24 fps)	Finger	1 × 60	–	N/R	Time-domain (N/R)	Yes	No	Sphygmomanometer (O) (Omron)	SBP = 3 ± N/R DBP = −1 ± N/R (ME)		MAPDs = 7.4 MAPDd = 9.1
Gao et al.^21^ (2016)	65 (25:40)	NTN	No	29 ± 7 N/R	PPG (green) from finger	Android phone (20 fps)	Finger	1 × 60	–	N/R	Time-domain, frequency-domain (N/R)	Yes	No	Sphygmomanometer (O) (A&D UA-767PBT)	SBP = 5.1 ± 4.3 DBP = 4.6 ± 4.3 (ME)	N/R	N/R
Gaurav et al.^22^ (2016)	3000 (N/R)	N/R	N/R	N/R N/R	PPG (red) from finger (databsase)	Samsung Galaxy Note 5 (100 fps)	N/R	N/R	–	N/R	Time-domain, derivative-based, heart rate variability-based (46)	No	No	From database	SBP = 4.47 ± 6.85 DBP = 3.21 ± 4.72 (MAE)	N/R	N/R
Plante et al.^23^ (2016)	85 (44:41)	NTN/HTN	N/R	56.6 ± 16.3 N/R	N/R	iPhone 5s and iPhone 6 (N/R)	Finger and chest	N/R	–	N/R	N/R	Yes	No	Sphygmomanometer (O) (Omron 907 and 907 XL)	SBP = 12.4 ± 10.5 DBP = 10.1 ± 8.1 (MAE)	*ρ*(SBP) = 0.44 *ρ*(DBP) = 0.41	N/R
Junior et al.^24^ (2016)	3 (1:2)	N/R	N/R	N/R N/R	PPG (red) from finger and PCG from chest	Samsung S4 (N/R)	Finger	N/R	–	N/R	PTT	No	Yes	N/R	N/R	N/R	N/R
Junior et al.^25^ (2015)	3 (1:2)	N/R	N/R	N/R N/R	PPG (red) from finger and PCG from chest	Samsung S4 (N/R)	Finger	N/R	–	N/R	PTT	No	Yes	N/R	N/R	N/R	N/R
Peng et al.^26^ (2015)	32 (7:25)	N/R	No	20–32 N/R	PCG from chest	Smartphone	Chest	13 × 60	–	N/R	Frequency-domain (36)	No	No	Finger BP cuff (Finometer MIDI, Model II)	SBP = 4.339 ± 6.121 DBP = 3.171 ± 4.471 (MAE)	*r*(SBP) = 0.707 *r*(DBP) = 0.712	N/R
Banerjee et al.^27^ (2015)	15 (N/R)	NTN/HTN	No	N/R N/R	PPG (Y from YCbCr) from finger	Nexus 5 (android) N/R	Finger	1 × 45	–	N/R	Time-domain, modeled signal (N/R)	No	No	Sphygmomanometer (O) (Omron)	N/R (i.e. given individually)	N/R	N/R
Visvanathan et al.^28^ (2014)	156 (N/R)	NTN/HTN/HoTN	N/R	21-42 N/R	PPG (red) from finger	iPhone 4 (30 fps)	Finger	1 × 23	–	N/R	Time-domain, frequency-domain(19)	Yes	No	Sphygmomanometer (N/R) (ETCOMM HC-502)	N/R	N/R	Acc_s_ = 98.12 Acc_d_ = 97.22 (classification)
Lamonaca et al.^29^ (2013)	5 (N/R)	N/R	N/R	N/R N/R	PPG (red) from finger	HTC Desire S (30 fps)	Finger	N/R	–	N/R	Time-domain (15)	No	No	Sphygmomanometer (O) (ABP SPACELABS 90207)	N/R	N/R	N/R
Visvanathan et al.^30^ (2013)	17 (N/R)	N/R	N/R	N/R N/R	PPG (red) from finger	iPhone 4 (N/R)	Finger	N/R	–	N/R	Time-domain (14)	Yes	No	Sphygmomanometer (N/R) (ETCOMM HC-502)	N/R	N/R	Acc_s_ = 98.7/100 Acc_d_ = 99.7/99.29 (Regression/SVM)
Li et al.^31^ (2013)	5 (N/R)	N/R	N/R	27 ± 6 N/R	PPG (green) from finger	Samsung Galaxy S4 i9500 (20 fps)	Finger	N/R	–	N/R	PWTT	No	Yes	Sphygmomanometer (O)	N/R	N/R	N/R
non-contact																	
Patil et al.^32^ (2019)	4 (N/R)	NTN	No	N/R N/R	PPG from facial video	Different smartphones (30 fps)	Face	10 × 10	40–60	Office room	PTT	No	N/R	Sphygmomanometer (O) (Omron)	N/R	*r*(SBP) = 0.64−0.94 *r*(DBP) = N/R	
Luo et al.^33^ (2019)	1328 (540:788)	NTN	Yes	46 ± 17 Diverse	TOI from facial video	iPhone 6+ front camera (30 fps)	Face	1 × 120	40–60	Study room, face illuminated	Time-domain (155)	Yes	No	Finger cuff (CNAP Monitor 500)	SBP = 0.39 ± 7.30 DBP = 0.20 ± 6.00 (ME)	*r*(SBP) = 0.67 *r*(DBP) = 0.47	Acc_s_ = 94.81 Acc_d_ = 95.71

*F:M* female:male, *N/R* not reported, *NTN* normotensive, *HTN* hypertensive, *HoTN* hypotensive, *PPG* photoplethysmography, *PCG* phonocardiogram, *IR* infrared, *TOI* transdermal optical imaging, *PTT* pulse transit time, *PWTT* pulse wave transit time, *BP* blood pressure, *A* auscultatory, *O* oscillometric, *SBP* systolic blood pressure, *DBP* diastolic blood pressure, *MAE* mean absolute error, *ME* mean error, *SD* standard deviation, *RMSE* root-mean-square error, *MAPD* mean absolute percentage deviation, *Accs* percent systolic accuracy, *Accd* percent diastolic accuracy.

Clinical evidence on the accuracy of the proposed BP measurement methods is a prerequisite for smartphones to be used as medical tools for BP monitoring. Therefore, a participant pool of an adequate size is vital for testing and validation. Moreover, the samples need to represent the entire spectrum of patients; for instance, a method that is validated with only male subjects is likely to be less accurate when tested on female subjects. Note that Fine et al.^34^ reported physiological differences between men and women can impact the photoplethysmography (PPG) waveform and other physiological signals. Furthermore, structural differences in the cardiovascular system, such as gender-dependent diameters of blood vessels or different average heart rates between genders^35,36^, and differing skin thickness between women and men, also have a direct impact on the PPG signal^37^. In this review, 14 of the 25 articles reported the ratio of female to male participants^9–12,15–17,19,21,23–26,33^.

### Inclusion of hypertensive subjects

Of the 25 articles, 10 included hypertensive subjects in their participant pool^9–12,14,17,19,23,27,28^. Three of these 10 articles also included hypotensive subjects along with normotensive and hypertensive subjects^9,12,28^. The sample sizes of these articles ranged between 13 and 965 subjects. Another three of 25 articles specified that only normotensive subjects were examined^21,32,33^. Their sample sizes range between 4 and 1328 subjects. The remaining 12 of 25 articles, with sample sizes between three and 3000 subjects, did not report on the hypertension status of their subjects^13,15,16,18,20,22,24–26,29–31^. In an article on PTT-based continuous BP estimation, Ding et al.^38^ obtained higher estimation accuracy of BP from a normotensive group compared to the results obtained from a hypertensive group. Consequently, clinical assessment of hypertension using only a smartphone requires sufficient clinical evidence of accuracy among hypertensive patients^6^. Therefore, it is important that articles on BP monitoring include hypertensive subjects in their testing and validation procedures.

### Subjects with comorbidities

Eleven of the publications provided information on subjects’ health^9–12,17,19,21,26,27,32,33^. Of these, seven included only subjects with no comorbidities other than hypertension^10,12,17,21,26,27,32^. One article excluded subjects with myocardial infarction of less than one week, pulmonary embolism, arrhythmia, and decompensated heart failure^9^. The remaining three articles provided more detailed statistics on their subjects’ cardiovascular diseases or corresponding risk factors^11,19,33^. Because clinical assessment of hypertension using only a smartphone requires clinical evidence of accuracy, it is critical to also include patients with comorbidities in the participant pool. This is particularly true for diseases or conditions that are related to hypertension or its risk factors, such as diabetes and obesity, which can cause cardiovascular diseases.

### Age distribution and skin tone

Aging leads to a multitude of anatomical and physiological changes that can alter the measured PPG signal^34,39^. Besides vascular changes such as increased collagen deposition and calcification that lead to an increased systolic BP^40^, the skin is known to become thinner with age^34^. This impacts the transmission of light through the skin, leading to changes in the PPG signal. Another factor that influences the acquired PPG signal and, by extension, PPG-based BP estimation, is skin tone. Different articles have reported a positive correlation between heart rate (HR)-error estimated from video-based PPG and skin tone scores according to the Fitzpatrick skin scores when using primarily green light^34,41^. Since HR is evaluated by measuring the distances between two consecutive systolic peaks in the PPG signal, also referred to as peak-to-peak interval, HR-error can be considered a quality measure of the PPG signal^41,42^. Another important finding by Shirbani et al.^41^ is the independence of the HR error from skin tone when recording the video-based PPG signal under brighter lighting conditions. As Mohapatra et al.^43^ state, a remedy for higher estimation errors with darker skin is the use of light sources at higher wavelengths, i.e., a combination of a green light source with a light source of a higher wavelength.

Fifteen of the 25 articles provided information about the subjects’ age distribution^9–12,16–21,23,26,28,31,33^, and only four out of 25 articles provided information about ethnicity and hence skin tone of the subjects^10,16,17,33^. This lack of information complicates the process of unbiased comparison of different BP estimation methods. If BP measurement using only a smartphone is to become a viable alternative to cuff-based BP monitoring, methods that deliver accurate results for the entire spectrum of patients regarding age and skin tone are needed. In the section below on algorithms, we discuss further how additional demographic information can leverage the performance of BP estimation methods.

### Acquiring PPG data from the finger

Among the 25 articles, we identified four types of data that correlate to BP and are measured at different body locations. These four data types are listed in Table 2. After the acquisition of the raw signal, different signal processing steps are applied, depending on the type of signal. Information from all articles with respect to the signal acquisition is summarized in Table 1. Different smartphone models are used for data acquisition in the analyzed articles. It is generally difficult to provide hard constraints regarding the smartphone sensor specifications. However, a sufficiently high camera frame rate is of particular importance, especially for the time-sensitive estimation of pulse transit time (PTT). The smartphone models and respective frame rates are listed in Table 1.

**Table 2:** Data types measured by a smartphone that are correlated with blood pressure.

Signal	Sensor	Method	Site
Photoplethysmography (PPG)			
Optical technique used to detect blood volume changes	Camera	Contact and non-contact	Finger, face
Transdermal Optical Imaging (TOI)			
Optical data-driven technique to detect changes in hemoglobin concentration	Camera	Non-contact	Face
Phonocardiography (PCG)			
The recording of the heart sounds	Microphone	Contact	Chest
Seismocardiography(SCG)			
The recording of body vibrations induced by the heart beat	Accelerometer	Contact	Chest

Reflection photoplethysmography is a technique used to non-invasively measure changes in blood flow and volume. If a smartphone is used as a photoplethysmograph, the subject’s finger is placed on the smartphone camera while the LED light is activated. This light is transmitted into the skin, where a certain amount is reflected. The reflected light that is measured with a photodiode (PD), contains static and pulsatile components, often referred to as direct current (DC) and alternating current (AC) components^44^. The terms originate in electronics and refer to the constant and alternating components of a signal. The AC component of the acquired PPG signal is a result of the volume changes of blood as it is pumped from the heart to the periphery, i.e., the measurement site. Consequently, the AC component carries meaningful information about the blood pulse wave. Thus, when acquiring the PPG signal from the finger, the objective is to extract the pulsatile component from reflected light. However, it has been shown that the AC component of a PPG signal accounts for only a small proportion of the total intensity, around 1%, of the intensity from the DC component, which poses a challenge to the signal processing^45^. Additionally, PPG signals measured with smartphone sensors are prone to motion and noise artifacts^46^. A low signal-to-noise ratio further complicates the extraction of meaningful information for BP estimation^20^. During signal collection, moving the finger, incorrectly placing the finger, or applying pressure can corrupt the PPG signal and hence the subsequent BP estimation.

Various techniques have been proposed in the literature to mitigate the negative effects of motion artifacts and incorrect finger placement on the PPG signal. As Lee et al.^47^ state, one approach to tackle such artifacts, based on the optical characteristics of tissue, can be the choice of light wavelength. Previous articles have shown that green is the light color least susceptible to motion artifacts^47^. Compared to the larger wavelengths of red and infrared that penetrate deeper into the tissue due to low absorption, the light at lower wavelengths such as green is strongly absorbed by hemoglobin, leading to a shallow penetration into the tissue^47^. Furthermore, motion artifacts tend to be more amplified in deeper sites^48^. Among the 21 articles that measure a PPG signal, nine extracted the green channel for BP estimation^9–13,17,19,21,31^, another nine extracted the red band^14,15,20,22,24,25,28–30^, and one article used the infrared (IR) component of the acquired signal^16^. Banerjee et al.^27^ used the Y component of the YC_R_C_B_ color space for BP estimation. One article did not report on any specific color channel extracted from the raw signal.

**Fig. 2:**
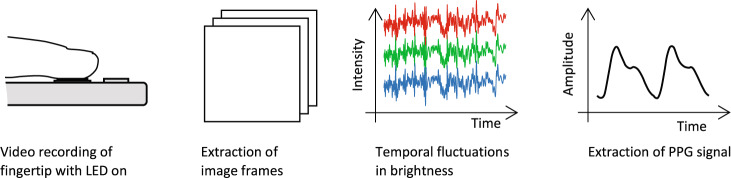
Construction of the PPG signal from finger-based video recording. After the video is recorded, a mean pixel brightness for each color channel (red, green, and blue) is computed over the entire frame. This results in a signal that reflects the temporal fluctuations in brightness for each color channel. Subsequently, a single-color channel (mostly green or red) is typically band-pass filtered to obtain the desired photoplethysmographic (PPG) signal. This schematic represents the general workflow and highlights commonalities among the different methods.

The most commonly applied method for obtaining a 1D PPG signal from a recorded video sequence is calculating the mean brightness of a particular color channel in each frame, leading to the desired signal. Subsequently, the raw signal is processed with the objective of extracting the meaningful variations in intensity caused by the periodic propagation of blood pulse waves through the arteries. The workflow of constructing a PPG signal from finger-based video recording is shown in Fig. 2. The contact-based PPG signal serves as a basis for BP estimation in 21 of the 23 articles that have proposed a contact-based approach. Of those 21 articles, four combined the PPG signal with heart sounds or seismocardiographic signals recorded at the chest to estimate BP^15,18,24,25^.

Schoettker et al.^12^ simultaneously recorded a raw optical signal from the patients’ fingers and continuous invasive BP before and during anesthesia. In each session, 10 recordings of one minute each were made. First, the pixels from the green channel in each frame were averaged, resulting in a PPG signal. Then, a quality index is given to each pulse wave according to its similarity to neighboring pulse waves. A weighted average of all pulse shapes based on the quality indices was generated and subsequently used as an acceptance criterion for all the recorded pulses. Pulses that did not meet the criteria were automatically rejected. Similarly, Visvanathan et al.^28,30^ adopted a finite-state machine-based approach to estimate the signal quality and to reject noisy video sequences^49^. Instead of reducing noise by assessing the signal quality, a group of articles simply applied a band-pass filter to reject unwanted frequencies^13,14,20,27^. The analyzed articles apply lower cutoff frequencies in the range of 0.5–4 Hz and upper cutoff frequencies in the range of 5–8 Hz. Besides band-pass filtering the raw signal, Datta et al.^20^ additionally applied mean subtraction and a baseline correction. In order to mitigate the effects of motion artifacts, a cycle selection was implemented. To this end, a histogram-based analysis on certain features was conducted, and cycles outside the most frequently occurring bin of a histogram were rejected^50^. Instead of just processing the acquired signal, Banerjee et al.^27^ modeled each cycle of the signal with a sum of two Gaussian functions. Then, root mean square error was used as a tool to assess the curve fitting quality. The modeled signal was subsequently used along with the original signal for BP estimation. The method outlined by Lamonaca et al.^29^ is the only one that identified a region of interest (ROI) for the contact-based signal acquisition. In each frame, a circled ROI based on the intensity histogram of the red component was identified. Then, an adaptive threshold was defined as the minimum brightness of the third quartile in the first *k* frames. This threshold was used to reject erroneous frames, i.e., frames that were too bright or too dark due to incorrect placement of the finger on the camera and LED. The PPG signal is computed as follows:

1 $${\rm {PPG}}[l]=\frac{{\rm {max}}(R)-R[l]}{{\rm {max}}(R)-{\rm {min}}(R)},$$

where the vector *R* contains the number of pixels with brightness higher than the adaptive threshold. The variable *l* denotes the *l*th element of the vector time, i.e., the *l*th frame. Junior et al.^24,25^ stressed the importance of locking the exposure time of the camera during measurements in order to prevent changes in the frame rate and image brightness during data acquisition. Since BP is estimated based on PTT, the temporal accuracy of the signal is pivotal, which requires precise reconstruction and delineation of the PPG signal in the time domain.

### Acquiring PPG and TOI data from the face

In non-contact PPG imaging, microvascular blood volume changes in tissues are extracted from a video sequence. In the literature, the term video PPG (vPPG) is typically referring to the methodology of extracting a PPG signal from a video, whereas the terms remote PPG (rPPG), non-contact PPG, and facial PPG are associated with the method of acquiring a PPG signal in a non-contact manner. Compared to contact-based PPG measurements, no direct contact between the subjects’ skin and sensor is required. This avoids the deformation of the arterial wall caused by pressing the finger on the camera, which leads to inaccurate measurements^45^. Furthermore, the contactless method has the advantage of not being limited to a single measurement site, which is the case for contact-based signal acquisition. This is particularly useful in cases where two or more signals from different sites are required for BP estimation. The possibility of recording multiple signals with the same sensor, i.e., the camera, renders a synchronization between signals obsolete. This is an asset for BP estimation based on PTT, which is outlined in the section on algorithms.

Measuring BP from a facial video sequence necessitates the detection of the face, the identification of regions of interest (ROIs), and an accurate tracking of these ROIs. Face detecion is usually performed in the first frame based on known algorithms such as Viola–Jones^51,52^. Similarly, existing algorithms such as the Kanade–Lucas–Tomasi feature tracker are applied to track specific ROIs in the subject’s face^51,52^. Patil et al.^32^ selected the forehead and right cheek as ROIs such that they cover the last branch of facial artery and the halfway of supratrochlear artery respectively. Luo et al.^33^ on the other hand, identified 17 ROIs with robust hemoglobin fluctuations across the face based on a spatiotemporal map of hemoglobin concentration. A major challenge to remote PPG signal acquisition is motion artifacts. The quality of the acquired PPG signal is significantly affected by movements of either the subject’s face or the camera^53^. This effect is further amplified by the use of a low-cost camera, such as a smartphone camera, with only ambient light^54^. Cho et al.^55^ hypothesized that motion artifacts are caused by an uneven reflection of light from the skin due to the curvature of the subject’s face and by the reflection of light from melanin and residual pigments, which is time-varying in the presence of movement. Based on this hypothesis, an adaptive noise cancellation algorithm based on recursive least square and the hue-saturation-intensity (HSI) model was applied^55^. Feng et al.^53^ proposed an approach that combines an enhanced facial tracking algorithm, an adaptive band-pass filtering method applied to the raw PPG signal, and automatic sorting of ICA output components based on a reference sine function^53^.

**Fig. 3:**
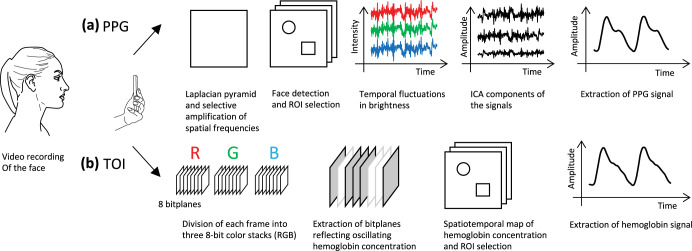
Construction of the PPG (a) and TOI (b) signals from a facial video. To obtain the PPG signal from the regions of interest, the frames are first processed to reduce noise and amplify the relevant information. Then, three signals are created per region of interest (ROI) by spatially averaging the pixel brightness of red, green, and blue over the entire ROI in each frame. In a last step, independent component analysis (ICA) is applied and the signal is band-pass filtered and smoothed to obtain the PPG signal. In the case of transdermal optical imaging (TOI), the information in each frame is divided into three color channels that are each comprised of an 8-bit color stack. Each bit of the stack constitutes a bitplane. Subsequently, bitplanes of the stack with characteristic fluctuations in one's are isolated using a machine learning model. With this data, a spatiotemporal map of hemoglobin concentration is created, regions of interest are identified and the hemoglobin concentration is spatially averaged in each frame to obtain a hemoglobin signal for each region of interest.

In our literature search, we found only two publications that are dedicated to contactless BP estimation with a smartphone^32,33^. However, the number of articles on rPPG-based BP estimation increases substantially if the constraint of using only a smartphone is removed. In the approach presented by Patil et al.^32^, several steps were needed to extract the desired PPG signal from the raw facial video. The approach is split into image processing and signal processing. As seen in the top row of Fig. 3, the image processing involves noise reduction by means of the image pyramid technique, detection of the face, selection of ROIs, and the amplification of spatial frequency bands that contain information about cardiovascular activity. The forehead and right cheek were selected as ROIs. After completion of the image processing steps, the PPG signal was acquired at both ROIs by spatially averaging the pixel intensities over the entire ROI. Then, an independent component analysis (ICA) was applied to compute the underlying source signals from the observed signals. Subsequently, the signals were band-pass filtered and smoothed by a moving average filter, which completed the process of extracting the intrinsic features of the PPG signals from the facial video.

Luo et al.^33^ first separated the information in each frame of the video by color channel as depicted in the bottom row of Fig. 3. In all channels—red, green, and blue—each frame is composed of an 8-bit color stack. Subsequently, all eight bitplanes were analyzed in terms of their temporal fluctuations in zeroes and ones. Based on a machine learning model, the bitplanes that fluctuated along with reference blood pressure, were isolated. These data were then used to identify 17 ROIs in the face with robust hemoglobin fluctuations. From these ROIs, the pixels were averaged in each frame, resulting in the desired hemoglobin signals. These averaged signals served as the basis for the subsequent estimation of BP.

### Acquiring PCG data from the chest

**Fig. 4:**
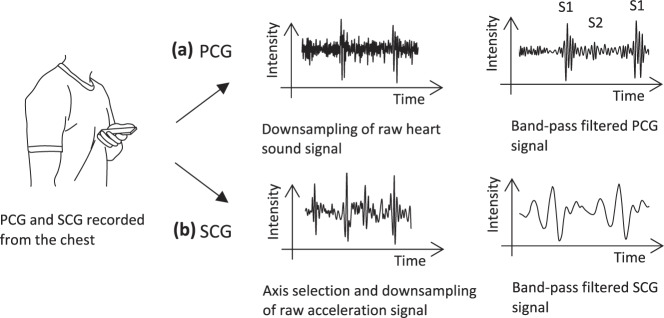
Construction of the phonocardiogram (PCG) and seismocardiogram (SCG) signal from recordings of heart sound and heart vibrations. The workflows depicted in a and b correspond to the PCG and SCG signal respectively. After the data are recorded, they are typically downsampled. Subsequently, a band-pass filter is applied. S1 and S2 are the two main heart sounds, representing the closure of the mitral and tricuspid valves, and the closure of the aortic and pulmonary valves, respectively.

The contraction and relaxation of the atria and ventricles, the valve movements, and the blood flow cause audible sounds^26^. During each cardiac cycle, there are two main heart sounds: S1, representing the closure of the mitral and tricuspid valves, and S2, representing the closure of the aortic and pulmonary valves^26^. The recording of the heart sounds leads to a signal that provides valuable information on the cardiac cycle. Nemcova et al.^15^ supported the subjects with an audio-visual feedback to ensure the correct placement of the sensors during measurements and thus improved the robustness and accuracy of the BP estimation. To acquire the PCG signal, the users were asked to place the smartphone’s bottom edge, where the microphone is integrated, perpendicularly to the mitral area of the heart. The raw PCG signal was then down-sampled from 48 to 1 kHz and band-pass filtered with an Infinite Impulse Response filter. Junior et al.^24,25^ recorded the heart sounds by placing the smartphone tightly on the chest. Similar to the approach of Nemcova et al.^15^, an audio–visual feedback was provided to support the user in correctly placing the sensors and thus to improve the signal quality. The raw PCG signal was down-sampled from 44.1 kHz to 900 Hz. Because BP estimation based on PTT requires synchronization between the two acquired signals, a synchronization procedure was introduced to ensure perfect alignment of the two signals. Peng et al.^26^ recorded the heart sound signal by connecting a stethoscope to the microphone on the earphone line. According to the authors, however, tests showed that the stethoscope is not needed in a quiet environment, and the heart sound signal can be recorded by pressing the smartphone’s microphone tightly to the subject’s chest. Since the PB estimation is based on the characteristics of the S2 wave, the stethoscope was placed at the right upper sternal border, where the S2 is stronger than that in other regions. The subjects underwent a measurement procedure of 13 min with an initial resting phase, a cold water stimulus, and a subsequent recovery phase. In order to reduce high-frequency noise, the signal was first filtered using a Butterworth low-pass filter with a cutoff frequency of 1000 Hz. An additional Butterworth high-pass filter with a cutoff frequency of 5 Hz was applied to remove baseline wandering. The general workflow of PCG signal acquisition is depicted in the top row of Fig. 4.

### Acquiring SCG data from the chest

Unlike phonocardiography, seismocardiography (SCG) does not rely on the heart sound signal but on the vibrations caused by the cardiac cycle. These vibrations can be measured using a smartphone’s accelerometer. According to Wang et al.^18^, the SCG is preferable to the PCG signal, as it actually captures the opening of the heart valves and is thus a better reference for the ejection of blood into the artery. Their article was the only one that incorporated an SCG signal into the BP estimation. They measured the SCG signal from the chest, as was the case for the acquisition of PCG signals.

The general workflow of PCG signal acquisition is depicted in the bottom row of Fig. 4.

### Pulse wave analysis methods

**Fig. 5:**
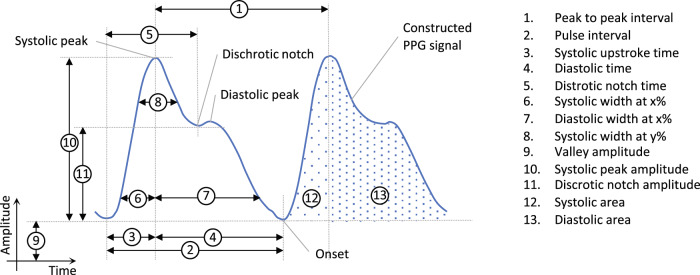
Commonly extracted features from the PPG signal for BP estimation. The figure displays a representative PPG signal during a time interval of two pulse waves, i.e., approximately two seconds.

In data-based methods, inherent characteristics of the pulse wave, i.e., the processed PPG signal, are collected as features and fed to a machine learning model. These features are commonly complemented by features from PGG derivatives and demographic information. Additionally, characteristics obtained from the frequency domain of the pulse wave signal can further improve estimation accuracy. The number of features fed into the model varies widely among different methods. Among the features depicted in Fig. 5, pulse interval, systolic upstroke time, and diastolic time are the most frequently used among the analyzed articles. In a few articles, principal component analysis (PCA) was applied to reduce the number of features and thus the computational burden. Baek et al.^13^ utilized a convolutional neural network to extract these features automatically. The literature reviewed presented a variety of different machine learning architectures, including artificial and convolutional neural networks, linear regression models, and support vector machines. In total, 15 articles based their BP-estimation on pulse wave analysis (Table 1). Schoettker et al.^12^ validated blood pressure measurements with the OptiBP smartphone app against reference auscultatory measurements. For parameter training, BP was measured invasively with a Philips IntelliVue MP50 monitor before and during the induction of general anesthesia. Simultaneously, the PPG signals were recorded using a smartphone via the contact method. The algorithm was validated using auscultatory BP values from a sphygmomanometer, as well as optical signals acquired by a smartphone. First, derivative-based features were extracted from the optical signal and nonlinearly combined using the trained parameters. Then, a corrective offset was added to the uncalibrated BP estimate. The approach presented by Schoettker et al.^12^ is the only data-based method among the analyzed articles that requires calibration. To reduce dependency on a particular sensor, Datta et al.^20^ omitted noise prone and amplitude dependent features or replaced them by ratios of PPG features. Instead of combining existing features, Lamonaca et al.^29^ extracted features according to two criteria: robustness to motion artifacts and minimization of error in the training and production phase of the artificial neural network (ANN). The ANN was set up and trained offline using the Multiparameter Intelligent Monitoring in Intensive Care database (MIMIC II) database. The algorithm was tested with the PPG data acquired via a smartphone and validated against the ABP spacelabs 90207 BP cuff. In the approach presented by Banerjee et al.^27^, a total of seven features were extracted based on the Maximal Information Coefficient after approximating the acquired PPG signal for each cycle by a sum of two Gaussian functions and removing outlier cycles based on *K*-Means clustering. A 2-Element Windkessel model representing the cardiovascular system in terms of resistance (*R*) and a capacitance (*C*) was set up, and the parameters *R* and *C* were estimated based on the Windkessel model and the PPG features using an ANN. Another source of features that several articles make use of is the frequency-domain representation of the PPG signal. Dey et al.^16^ for example, extracted a total of 233 features in the time and frequency domain, including features from the first to fourth derivative of the PPG signal and from the upslope and downslope deviation curve. A Lasso regression model was applied first to estimate diastolic BP. Along with the other features, this estimate was then incorporated into another Lasso regression model to estimate systolic BP. Subsequently, the data set was partitioned according to physiological and demographic parameters (i.e., age, gender, and BMI), and separate Lasso models were trained on each partition. Gao et al.^21^ applied a discrete wavelet transform (DWT) to the collected PPG and fed thousands of DWT coefficients, combined with the systolic upstroke time and the diastolic time, gender, and age, into a linear support vector machine for feature extraction. Subsequently, a nonlinear support vector machine was used to train the model. A support vector regression machine was then used to predict BP from the given data. Gaurav et al.^22^ extracted features for BP estimation not only from the PPG signal and acceleration plethysmogram (APG) waveform (the second derivative of the PPG signal), but also from heart-rate variability. A weighted average of three ANNs for systolic and diastolic BP was used to estimate each separately. The approach presented by Luo et al.^33^ was the only one where the extracted features are largely based on the TOI signal instead of the PPG signal. In this method, 126 features were obtained from facial transdermal blood flow signals. In total, 155 features were extracted. These were grouped under categories of pulse amplitude, heart rate band pulse amplitude, pulse rate, pulse rate variability, PTT, pulse shape, and pulse energy. PCA was conducted to reduce the number of features to 30 decorrelated eigenvectors that were fed into a multilayered perception machine-learning algorithm.

### Wave propagation methods

**Fig. 6:**
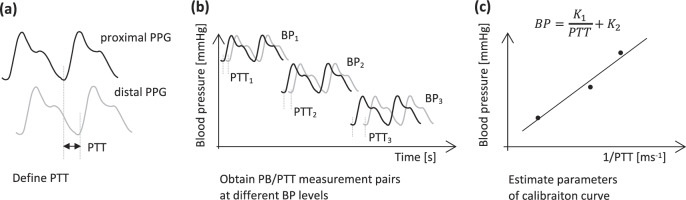
Workflow of PTT-based BP estimation. PTT stands for pulse transit time, PCG for Phonocardiogram, and BP for blood pressure. First, PTT needs to be defined based on the available signals, where different combinations can be used (**a**). Then, multiple measurement pairs of PTT and BP need to be obtained (**b**). With these measurements, the unknown parameters of the calibration curve can be estimated (**c**).

Pulse transit time is defined as the time needed for a pulse pressure wave to propagate a certain length through the arterial tree between a proximal and a distal measurement site, where the proximal site is closer to the heart^32^. It is noteworthy that compared to pulse wave analysis, BP estimation using PTT requires measurements to be taken at two sites simultaneously. Together with the pre-ejection period (PEP), PTT sums up to the pulse arrival time (PAT), as follows:

2 $${{{\rm{PAT}}\; =\; {\rm{PEP}}\; +\; {\rm{PTT}}}},$$

where PEP is the time difference between the ECG R-wave and the aortic valve opening, which is captured in the PCG and SCG signal^18,56^. According to Wang et al.^18^ and Zhang et al.^57^, PAT is not an accurate measure to estimate BP since it includes PEP which is influenced by the nervous system activity and is variable up to tens of milliseconds. The PPG waveform foot (also referred to as the diastolic point, onset, or minimum of the PPG signal) represents the preferred feature for the estimation of PTT^58^. Among the analyzed articles, Nemcova et al.^15^ calculated PTT as the time difference between S1 peak of the PCG signal and the systolic peak (maximum) of the PPG signal, and also in Patil et al.^32^ the peaks of the proximal and distal PPG signal are used to estimate PTT. Wang et al.^18^ and Junior et al.^24,25^ used the onset of the PPG wave for PTT estimation. PTT is known to be inversely correlated with systolic and diastolic BP.^58^ This correlation serves as a basis for PTT-related BP monitoring. In the literature, the mathematical relationship from PTT to BP is typically defined using physical models or empirical regression models.^58^ Nemcova et al.^15^ employed a linear model in the form BP = *a*⋅PTT + *b* to relate PTT to systolic BP. The model depicted in the top right corner of Fig. 6 was used by Wang et al.^18^ and was also recommended by Mukkamala et al.^58^, who examined different models in their article. Estimating BP from PTT requires a calibration of PTT in msec to BP in mmHg^59^. To this end, multiple simultaneous measurements of PTT and reference BP need to be obtained over a wide range of BP values to estimate the unknown parameters of the calibration curve, i.e., the applied model^58^. This process is depicted in Fig. 6. Generally, the more measurements that are available for the calibration, the more accurate the fitted model. However, the number of available measurement pairs needs to be equal or larger than the number of unknown parameters. This calibration curve between PTT and BP is subject-specific and changes over time, which necessitates a frequent and individual re-calibration^58^. In sum, PTT-based BP measurement is based on the following steps: measurement of waveforms at proximal and distal sites, PTT estimation from the waveforms, and calibration of PTT to BP based on several measurement pairs of PTT and BP^58^. Five of the 25 articles based their BP estimation on PTT. In Nemcova et al.^15^, the quantities ejection time (time difference between S1 and S2), heart rate, age, height, weight, and an individual calibration constant (*q*) were required to calculate BP. Systolic BP was calculated based on q and PTT. Diastolic BP was measured using the additional quantities systolic BP, stroke volume, body surface area, and pulse BP as well as age, height, and weight. Instead of using the microphone, Wang et al.^18^ collected data from the camera and the accelerometer subsystems. Subsequently, the temporal difference between the aortic valve opening (AO) from the chest SCG and the onset of the finger PPG was computed to obtain the desired PTT. Calculating BP from the PTT required an individualized calibration for each subject. Junior et al.^24,25^ measured the PAT at the proximal point using a smartphone’s microphone as a low-cost phonocardiogram. PAT at the distal point was computed from the PPG signal. PTT was then calculated using the detected fiducial points from the ensemble averaged signals. As the only approach that acquired the PPG signals in a non-contact fashion, Patil et al.^32^ calculated the PTT from the temporal differences of local maxima between the two PPG signals from the forehead and right cheek. It was then shown that a strong correlation exists between the systolic BP measurements and the acquired PTT. However, no absolute BP numbers were estimated, and no information on diastolic BP was given.

### Further methods for blood pressure estimation

In addition to the 15 articles that estimated BP based on waveform analysis and another five that calculated the PTT for BP estimation, five articles proposed methods that differ from the two common approaches. Three of these articles are noteworthy. Yamakoshi et al.^10^ based their BP estimation on the hemodynamic Ohm’s law, i.e., the cardiac output (CO) multiplied by the total peripheral resistance (TPR) results in mean BP. CO and TPR are linearly correlated with pulse rate (PR) and mNPV, respectively, which can both be measured using smartphone sensors. However, this correlation only holds true for subjects who are in a resting position and inactive. With an initial calibration, both systolic and diastolic BP values can be calculated based on Ohm’s law and the assumption of approximate proportionality outlined above. Another approach was presented by Matsumara et al.^17^ who derived an exponential transformation of the linear polynomial equation based on heart rate (HR) and modified normalized pulse volume (mNPV) to calculate BP. Peng et al.^26^ estimated BP using only heart sound signals. Compared to the approaches outlined in the previous sections in which heart sounds were combined with PPG signals to calculate PTT, this method took advantage of the correlation between the second heart sound (S2) and BP. First, S2 was identified using the Shannon energy envelope of the acquired signal. Then, features were extracted from the frequency domain of the heart sound signal, i.e., its sequences around S2. A support vector machine (SVM) was then used to estimate systolic, diastolic, and mean BP.

### Incorporation of demographic information

As outlined in the section on the subjects, demographic information provides valuable information on a multitude of anatomical and physiological characteristics that can influence the signals acquired from subjects’ bodies, and hence the subsequent BP estimation. Research has also shown that demographic and physiological characterization improves BP prediction^16^. However, as Mukkamala et al.^60^ state, demographics alone are also known to correlate with BP. Consequently, if the prediction accuracy depends largely on the additional demographic information, the BP measurement method itself might not add much value. In this review, only 11 of the 25 analyzed articles included demographic information such as gender, age, or weight in their models (Table 1).

### Calibration

The methods found in the literature that propose BP measurement with only a smartphone can be categorized into cuff-calibrated and calibration-free approaches^60^. Typically, approaches based on waveform analysis allow a calibration-free BP estimation, whereas the methods based on PTT require calibration (Table 1). Cuff-calibrated methods only track BP changes and rely on inter- and intraindividual BP variations for the calibration and evaluation^60^. Possibilities to invoke changes in BP during measurement include exercise, cold pressor test, mental test, and drug effects, or variations that occur naturally during daily life, such as rest, stress, meal, activity^60^. However, recording data that contains BP changes in subjects can be difficult. First, most methods that propose BP estimation based on PTT require a second measurement to be taken at the chest, which is very susceptible to motion artifacts. Thus, exercising is not well suited to invoke BP changes for the evaluation of such methods. Second, interventions for changing the subject’s BP can pose a risk to some individuals^60^. Eight of the 25 articles reviewed required initial calibration to generate absolute BP values^9,10,12,15,18,24,25,31^. The need for calibration raises the question of practicality. The main advantage of BP monitoring with only a smartphone is the reduction in the dependency of patients on clinical care, allowing for cost-effective, unobtrusive, and remote BP monitoring throughout the day. The necessity for frequent or at least initial calibration undermines this advantage. Furthermore, there is neither consensus on the frequency of re-calibrations nor is it clear whether such re-calibrations attain the required level of accuracy when performed by patients^11^. The need for at least an initial calibration is an inherent disadvantage of PTT-based BP estimation methods.

### Evaluation protocol

For the clinical use of medical devices, an evaluation based on established validation protocols is a must. The evaluation of BP measuring devices constitutes strict guidelines on the reference BP measurement and validation procedures, including specifics about the participant pool, the chronology of measurements, the reference BP monitors, and evaluation metrics. Accurate measurement of blood pressure is pivotal to the reliable diagnosis and efficient treatment of hypertension^61^. Therefore, only BP measuring devices, which have been successfully validated by using an established protocol, should be used^62^. In the past, several protocols developed by scientific organizations have been used for the clinical validation of electronic BP monitors^62^. In 2018, an international initiative was taken by the US Association for the Advancement of Medical Instrumentation (AAMI), the British Hypertension Society, the European Society of Hypertension (ESH) Working Group on Blood pressure agreed to develop a universal standard for device validation^63^. This universal standard is now in use and referred to as AAMI/ESH/ISO Universal Standard (ISO 81060-2:2018). Key aspects of the AAMI/ESH/ISO Universal Standard (ISO 81060-2:2018) are the validation study efficacy measure, sample size, cuff-size stratified subgroups, general population and special populations studies, method for BP data collection, reference BP measurement and validation procedure, validation criteria and reporting, validation of other BP monitors, and quality and reliability of validation study reports^63^.

To date, there is no validation protocol available for BP measurement using only a smartphone. The existing validation protocols are not intended for cuffless BP measurement methods^60^. First of all, PTT-based BP estimation methods require several measurements at different BP levels for calibration, whereas the existing protocols, i.e., the AAMI/ESH/ISO Universal Standard (ISO 81060-2:2018), require stable BP throughout the measurement procedure. In case of BP estimation based on remote PPG, clear guidelines for data acquisition are needed, including illumination of the face and robustness in the presence of motion artifacts. More significantly, the current gold standard (auscultation) only delivers intermittent BP measurements and is thus not capable of assessing the accuracy of continuous BP readings. Therefore, invasive BP measurements are needed to verify the capability of the proposed methods to follow quick BP changes^64^.

The reference BP measurement and validation procedures used for performance assessment vary widely among the analyzed articles. A total of four articles based their validation procedure on existing protocols from AAMI, ESH, and ISO^9,11,12,19^. In four articles, a measurement procedure is used that induces changes in BP. Peng et al.^26^ caused a BP perturbation by exercising the patients during measurements. Baek et al.^13^ measured subjects’ BP during resting, sleeping, and exercising while Junior et al.^24,25^ asked the subjects to perform the Valsalva–Weber maneuver^65^ to induce changes in BP during measurement. As discussed above, in the Subjects subsection, the proposed methods are evaluated using a wide range of sample sizes. Furthermore, different evaluation metrics are applied to assess the accuracy of the BP estimation.

### Gold standard

According to the AAMI/ESH/ISO Universal Standard (ISO 81060-2:2018)^63^, reference BP measurements need to be performed with mercury sphygmomanometers or accurate non-mercury devices that were validated to an established international standard. As Alpert et al.^66^ state, auscultation requires a lot of training and maintenance of quality control to avoid inaccuracies. Furthermore, mercury is being banned in a growing number of countries due to presumed environmental and health effects^66^. Therefore, oscillometric devices have become the clinical standard^66^. Of the 25 articles, 18 used a sphygmomanometer for performance assessment. Of these 18 articles, 4 performed the reference BP measurement with an auscultatory (mercury) sphygmomanometer^9,12,15,16^, another ten used an oscillometric sphygmomanometer^11,18–21,23,27,29,31,32^, and four reported the use of a sphygmomanometer without further information on the type^10,17,28,30^. In one article, a Finometer with a finger cuff was used to measure the reference BP^26^. Luo et al.^33^ measured reference BP with a continuous BP monitor based on a finger cuff that was approved for medical use by the US Food and Drug Administration (FDA). Four articles did not provide any information on the reference BP values used^13,14,24,25^. Another article relied on the MIMIC II database for comparison^22^.

### Evaluation metrics

The publications analyzed in this review utilized different methods of performance assessment. In 16 of the articles, the accuracy of the proposed BP estimation was determined by mean (absolute) Bland–Altman agreement for systolic and diastolic BP. In 11 of the articles, a correlation coefficient, either Pearson or Spearman, was presented as the accuracy measure. Three articles, including the two that classified BP values into bins, gave percent accuracy. Five articles did not report the accuracy of their method compared to a reference BP (Table 1). Six articles satisfied pass-fail criterion 1 of the AAMI/ESH/ISO 81060-2:2018 norm, i.e., mean systolic and diastolic BP difference and their standard deviation smaller or equal to 5 ± 8 mmHg^9,10,12,22,26,33^. However, it is important to note that passing criterion 1 alone is not sufficient for successful validation of the tested method, as all other requirements of the protocol need to be fulfilled. The validation information from all of the articles is summarized in Table 1.

## Discussion

The purpose of this review is to assess the current state in the literature and guide future research dedicated to BP monitoring using only a smartphone. To that end, we conducted an extensive literature search to identify previous studies in the field of BP measurement with only a smartphone. Although numerous articles propose cuffless BP monitoring methods involving a smartphone, a majority incorporates additional devices, such as wristbands, chest bands, ECG sensors, or a second smartphone. It is particularly striking that there have been such a small number of studies focusing on non-contact smartphone-based BP estimation. Most articles dedicated to noncontact BP estimation rely on an advanced camera setup.

The detection, diagnosis, and monitoring of hypertension as performed today is subject to several limitations. First, the worldwide prevalence of hypertension, paired with a lack of clear and distinctive symptoms, lowers the chance of early detection. Second, BP monitoring is usually conducted in clinical settings. However, according to O’Brien et al.^67^, BP monitoring in an ambulatory setting has several advantages over clinical BP monitoring. These include the possibility of acquiring a BP profile in the patient’s usual daily environment, a larger number of readings taken over a longer time period, and assessment of BP variability over 24 h. Furthermore, ambulatory BP monitoring is a stronger predictor of cardiovascular morbidity and mortality than clinical BP monitoring. Third, once hypertension is detected, it is most commonly monitored in an ambulatory setting using a cuff-based BP monitor. However, such cuffs are obtrusive and uncomfortable to wear and deliver only intermittent BP measurements. Given the prevalence of hypertension, the deployment of BP cuffs on such a large scale does not seem realistic in terms of cost and feasibility. Measuring BP with a smartphone has great potential for alleviating the current limitations in BP monitoring. As a device with unparalleled ubiquity, it has the potential to serve as a cost-effective and unobtrusive BP monitor, paving the way for long-term ambulatory BP monitoring on a large scale. Two main facets of BP estimation with only a smartphone were identified in the literature: video-based non-contact methods where all the information is extracted from a facial video sequence and contact methods that require direct contact between the measurement site and the sensor. As seen in our analysis, non-contact methods based on facial videos have the advantage of not being limited to a single measurement site, making them less susceptible to physiological irregularities. The possibility of acquiring signals simultaneously from multiple ROIs with the same sensor, i.e., the camera, is another asset to the non-contact approach, as it enables the estimation of PTT without the need of an additional signal from another sensor. Furthermore, acquiring the signal in a contactless manner avoids the deformation of the arterial wall caused by pressing the finger on the camera, which can lead to inaccurate measurements due to changes in the optical properties of the tissue induced by deformation, or due to a hampered blood circulation in the arteries and capillaries underneath the skin. The forgoing analysis of the existing literature has shown that BP monitoring with only a smartphone is not ready for clinical use yet. First, only six of the 25 studies satisfied the accuracy threshold for clinical acceptance. Second, clinical validation with a clear protocol that sets strict guidelines for experimental design remains missing. We identified a large variety of experiment designs and participant pools, which complicates the comparison of the performance of different approaches. Furthermore, testing and validating the methods with small sample sizes is an unreliable performance indicator, as results may differ considerably when tested on larger samples. Third, most experiments were conducted in lab environments that do not represent real-life situations. Based on our findings throughout this review, we recommend the following:

- Future research must pay more attention to the underlying data that they use. First, the participant sample sizes should be chosen in accordance with the guidelines provided in established validation protocols. For an AAMI/ESH/ISO validation study, at least 85 subjects are required. Second, the inclusion and exclusion criteria should be clearly communicated. Third, the participant sample must include a broad spectrum of subjects representative of a possible target population. This includes subjects with different hypertensive status, skin tone, age, gender, and comorbidities.
- Incorporating demographic information into BP estimation models can improve accuracy. Future articles should take advantage of such information that can easily be collected from the subjects. Especially in data-driven methods, prior research has shown that categorizing the dataset according to physiological and demographic information improves estimation accuracy.
- Investigating pregnant populations is particularly important, as detecting hypertension at an early stage and relevant measures for it can slow down or prevent disease progression^19^. The same holds for pediatric populations.
- In order to be applicable in clinical use, future articles need to validate their methods in real-world environments beyond a fixed lab setup. Non-contact BP measurement methods need to be robust to environmental changes, such as alternating lighting conditions and motion artifacts. The same holds true for contact methods susceptible to noise, given that they rely on heart sound signals to estimate BP.
- Future research must scrutinize the calibration of smartphones for BP monitoring. It is important to know how accuracy evolves over time, i.e., how frequently a smartphone needs to be calibrated and whether a calibration can be carried out by the patients themselves.

Our literature search has shown a clear increase in research activity in the field of BP estimation with only a smartphone over the last decade. Methods of estimating BP through pulse wave analysis have especially gained momentum. Technical advancements in smartphone cameras and processors and an ever-growing demand for video-based communication technology have made an imminent breakthrough in non-contact BP monitoring likely. Weaving BP monitoring unobtrusively into our everyday lives as we face screens throughout the day has the potential for cost-effective early detection and large-scale monitoring of hypertension. Contact-based BP measurement, on the other hand, may prove useful in settings where screen time is considerably lower. Despite these promising tendencies, the proposed approaches are not yet ready for clinical validation. Indeed, not a single smartphone application for BP measurement has been approved for medical use yet. Of the 25 articles, six satisfied the pass-fail criterion 1 of the AAMI/ESH/ISO 81060-2:2018 norm, i.e., mean systolic and diastolic BP difference and their standard deviation smaller or equal to 5 ± 8 mmHg. Nonetheless, further research is necessary to improve the accuracy and robustness of BP estimation using only a smartphone. Moreover, as there is no established validation protocol available for smartphone-based BP measurement technologies at present, establishing an internationally accepted protocol for clinical validation is a pivotal step towards the clinical use of such technology. Data security is another issue that needs to be dealt with once the technology is ready for use. In conclusion, smartphone-based BP monitoring has the potential to make regular long-term BP monitoring accessible to a large section of society, thus contributing substantially to the immense challenge of detection, diagnosis, and monitoring of hypertension. Much further study is needed, however, to make this a reality.

### Reporting summary

Further information on research design is available in the Nature Research Reporting Summary linked to this article.

## Supplementary information

Reporting Summary

## Data Availability

The authors declare that all data supporting the findings of this study are available within the paper.
